# Application of Direct Immersion Solid-Phase Microextraction (DI-SPME) for Understanding Biological Changes of Mediterranean Fruit Fly (*Ceratitis capitata*) During Mating Procedures

**DOI:** 10.3390/molecules23112951

**Published:** 2018-11-12

**Authors:** Hasan Al-Khshemawee, Xin Du, Manjree Agarwal, Jeong Oh Yang, Yong Lin Ren

**Affiliations:** 1 School of Veterinary and Life Science, Murdoch University, 90 South St., Murdoch, WA 6150, Australia; hasan_hadi1984@yahoo.com (H.A.-K.); b.du@murdoch.edu.au (X.D.); 2 College of Agriculture, Wasit University, Wasit 120, Iraq; 3 Plant Quarantine Technology Centre, Animal and Plant Quarantine Agency (APQA), Gimcheon 39660, Korea; joyang12@korea.kr

**Keywords:** DI-SPME, GC-MS, Mediterranean fruit fly, extraction solvent, metabolites

## Abstract

Samples from three different mating stages (before, during and after mating) of the Mediterranean fruit fly *Ceratitis capitata* were used in this experiment. Samples obtained from whole insects were subjected to extraction with the two mixtures of solvents (acetonitrile/water (A) and methanol/acetonitrile/water (B)) and a comparative study of the extractions using the different solvents was performed. Direct immersion-solid phase microextraction (DI-SPME) was employed, followed by gas chromatographic-mass spectrometry analyses (GC/MS) for the collection, separation and identification of compounds. The method was validated by testing its sensitivity, linearity and reproducibility. The main compounds identified in the three different mating stages were ethyl glycolate, α-farnesene, decanoic acid octyl ester, 2,6,10,15-tetramethylheptadecane, 11-tricosene, 9,12-(*Z*,*Z*)-octadecadienoic acid, methyl stearate, 9-(*Z*)-tricosene, 9,11-didehydro-lumisterol acetate; 1,54-dibromotetrapentacontane, 9-(*Z*)-hexadecenoic acid hexadecyl ester, 9-(*E*)-octadecenoic acid and 9-(*Z*)-hexadecenoic acid octadecyl ester. The novel findings indicated that compound compositions were not significantly different before and during mating. However, new chemical compounds were generated after mating, such as 1-iodododecane, 9-(*Z*)-tricosene and 11,13-dimethyl-12-tetradecen-1-acetate which were extracted with both (A) and (B) and dodecanoic acid, (*Z*)-oleic acid, octadecanoic acid and hentriacontane which were extracted with (A) and ethyl glycolate, 9-hexadecenoic acid hexadecyl ester, palmitoleic acid and 9-(*E*)-octadecenoic acid, which were extracted with solvent (B). This study has demonstrated that DI-SPME is useful in quantitative insect metabolomics by determining changes in the metabolic compounds in response to mating periods. DI-SPME chemical extraction technology might offer analysis of metabolites that could potentially enhance our understanding on the evolution of the medfly.

## 1. Introduction

The developed analytical methods for the analysis of volatile and non-volatile compounds are increasingly being used as tools for the study of plant chemistry and the evolution of insect–plant interactions [1]. The development of sample preparation and extraction methodologies is one of the main challenges for metabolism studies [2] and has an enormous impact on the quality of the data. Biological samples should be unbiased and nonselective [3]. Solid-phase microextraction (SPME) has been used for rapid sample preparation and provides an efficient method to detect chemicals in detection and separation systems [3,4]. The extraction of samples can be performed using two methods. In the first method, headspace SPME (HS-SPME), the polymeric film is exposed to the gas phase that adsorbs the volatiles in the headspace of the liquid, gas or gaseous samples. The second method is direct immersion (DI-SPME), in which the fiber is directly immersed in a small volume of the liquid-extracted sample [5,6,7]. After the sample matrix and SPME coating achieves equilibrium, the extracted SPME is inserted into a gas chromatograph-mass spectrometer (GC-MS) for thermal desorption or into a desorption solvent for coupling with liquid chromatography (LC-MS) [8]. In addition, SPME has been used on environmental samples for the extraction of volatile organic compounds and has been a focus of interest in analytical biology, as well as pharmaceutical and food studies [4]. Some of the compounds function as species-specific signals, i.e., pheromones that provide intraspecific information [9]. Several studies have investigated the volatile pheromonal emissions released by the medfly as a potential source for an effective virgin female attractant. This would be useful such as an attractant might also find use in female annihilation programs and in mating disruption studies. The mating behavior of the mature male medfly, is also associated with the release of pheromonal volatiles attractive to the female fly [10]. Studies further describe this calling process and suggested that several abdominal glands present in the males were involved in production and released of the pheromone mixture [11,12]. A more extensive list of the biological activity of medfly including (f)-2-hexenoic acid, linalool, geranyl acetate, 2,3- and 2,5-dimethylpyrazines was reported by Jang et al. [12] and identification by Heath et al. [13]. Also, Baker et al. [14] have monitored the release of three of the major male medfly emission components which are ethyl (f)-3-hexenoate, geranyl acetate, and α-farnesene. In fruit flies, long-chain hydrocarbons on the adult fly cuticle are perceived by other flies over a short distance. Several studies have investigated the role of these compounds in chemical communication in the fruit fly [15]. Recently, SPME has been combined with capillary electrophoresis and liquid chromatography, and used for various biological samples, e.g., plasma and urine [16]. 

The development of analytical technology with powerful quantitative and qualitative capabilities, as well as high specificity, is required for the study of metabolic samples. This study investigates the feasibility of using DI-SPME high-resolution metabolism for profiling of fruit fly tissues at different stages of adulthood. Headspace-SPME (HS-SMPE) has been reported to be selective for volatile analyses, and is highly sensitive to volatile chemicals [17,18,19]. This approach enables SPME to identify substances with poor chromatographic behavior, thermal instability, or high reactivity [20]. Here, to ensure a high degree of sensitivity and chemical specificity, SPME with a GC-MS was used to capture metabolites [21]. The potential uses of DI-SPME for extracted insect samples were tested using statistical analysis to detect changes in the extraction samples before, during and after mating. The growth of metabolic and chemical analyses involving these low-dimensional score plots necessitates the use of quantitative statistical measures to describe significant differences between experimental groups such as PCA/PLS-DA score plots [22]. The PLS-DA is the first application of multivariate statistical methods for classification by ambient ionization but these methods have been applied previously to other MS imaging methods [23]. Principal component analysis has been used successfully as a multivariate statistical process control tool for detecting differences in processes with highly correlated variables [24]. Finally, DI-SPME was used in response to the need for the acquisition of representative metabolism data and for a better understanding of the encountered effects of extract samples. 

## 2. Results and Discussion

The precision of DI-SPME was tested using biological sources and analysis of variation, to determine the analytical variability of the data generated when adult flies were sampled at different stages. In this experiment, the DI-SPME samples of mature medfly adults at three different mating stages (before, during and after), were analyzed using a GC-MS. To examine the effectiveness of DI- SPME, two different solvent extractions were used to compare the DI-SPME, which indicated quantitative and qualitative differences between these solvents in the type and peak areas of compounds. For further testing of DI-SPME, a GC-MS was used to compare the composition of two extracts solvents after directly immersing the SPME fiber in the extract. Comparison of these compound profiles revealed that DI-SPME had higher levels of the lighter chemicals and lower levels of ponderous chemicals. Firstly, the choice of the sealing and desorption time was carried out by fixing the time (2, 4, 8 and 16 h of sealing times). The best results were obtained with the recently developed 50/30 μm Carboxen/DVB/PDMS and, thus, 16 h sealing time was selected for further method development.

Overall, DI-SPME detected 110 compounds using the acetonitrile/water solvent and 86 compounds using the methanol/acetonitrile/water solvent at three different stages. In the first solvent extraction, 47, 26 and 37 compounds were identified from samples taken before, during and after mating, respectively. In the second solvent, 33, 31 and 22 compounds were identified from the samples taken before, during and after mating, respectively. The method has developed a strategy for rapid comparison of non-processed MS data files. To explain the differences between the samples, the method includes the following: baseline correction; alignment; time window determinations; alternating regression; PLS-DA. The identification of the retention time, the retention index, and mass spectral, MS structurally ordered separation windows in the chromatograms. For understanding the trends in analytical variability of our data set generated when different sides of solvents were sampled, chemically and functionally distinct metabolites were tentatively identified with retention index, the aid of mass spectral similarity, injection of authentic standards (C_7_-C_30_), and structurally ordered separations. The results showed significant correlations between metabolite molecular weight, the retention index and metabolites. The main compounds identified were ethyl glycolate, α-farnesene; decanoic acid octyl ester; 2,6,10,15-tetramethylheptadecane, 11-tricosene, 9,12-(*Z*,*Z*)-octadecadienoic acid, methyl stearate; 9-(*Z*)-tricosene, 9,11-didehydrolumisterol acetate; 1,54-dibromotetrapentacontane, 9-(*Z*)-hexadecenoic acid hexadecyl ester, 9-(*E*)-octadecenoic acid, and 9-(*Z*)-hexadecenoic acid octadecyl ester, (Table 1 and Table 2). 

For some compounds, there were significant differences observed between samples collected at different stages. In the first solvent extraction, tetracosane; diethyldecyloxyborane, 9-(*Z*)-tricosene, hexacosane; 9-(*E*)-octadecenoic acid, 1,54-dibromotetrapentacontane, *trans*-13-octadecenoic acid, 2-methyloctacosane, 11,13-dimethyl-12-tetradecen-1-ol acetate; TBDMS-1-eicosanol; octatriacontyl pentafluoropropionate; 1-iodododecane, octadecanoic acid, supraene and hentriacontane were significantly different between the collection periods (Table 1). In the second extraction solvent, 9-hexadecenoic acid pyrrolidide; diclofop-methyl; 1-piperidin-1-yl-hexadecan-1-one; stigmasta-3,5-diene; ethyl glycolate; 1,2-dihydro-2,2,4-trimethylquinoline, palmitoleic acid, 9,12-(*Z*,*Z*)-octadecadienoic acid, methyl stearate and trimesitylborane were identified (Table 2). Principal component analysis (PCA), sparse partial least squares-discriminant analysis (sPLS-DA), and heat map and ANOVA analyses were used in these experiments. PCA visualizes both the covariance and correlation between the metabolites and the modeled class designation. Thereby the PCA-plot helps to identify statistically significant and potentially biochemically significant metabolites, based both on contributions to the model and their reliability. An extension of PCA, the sPLS-DA-plot, is applied to compare the outcome of multiple classification models compared to a common reference, e.g. control. 

**Figure 1:**
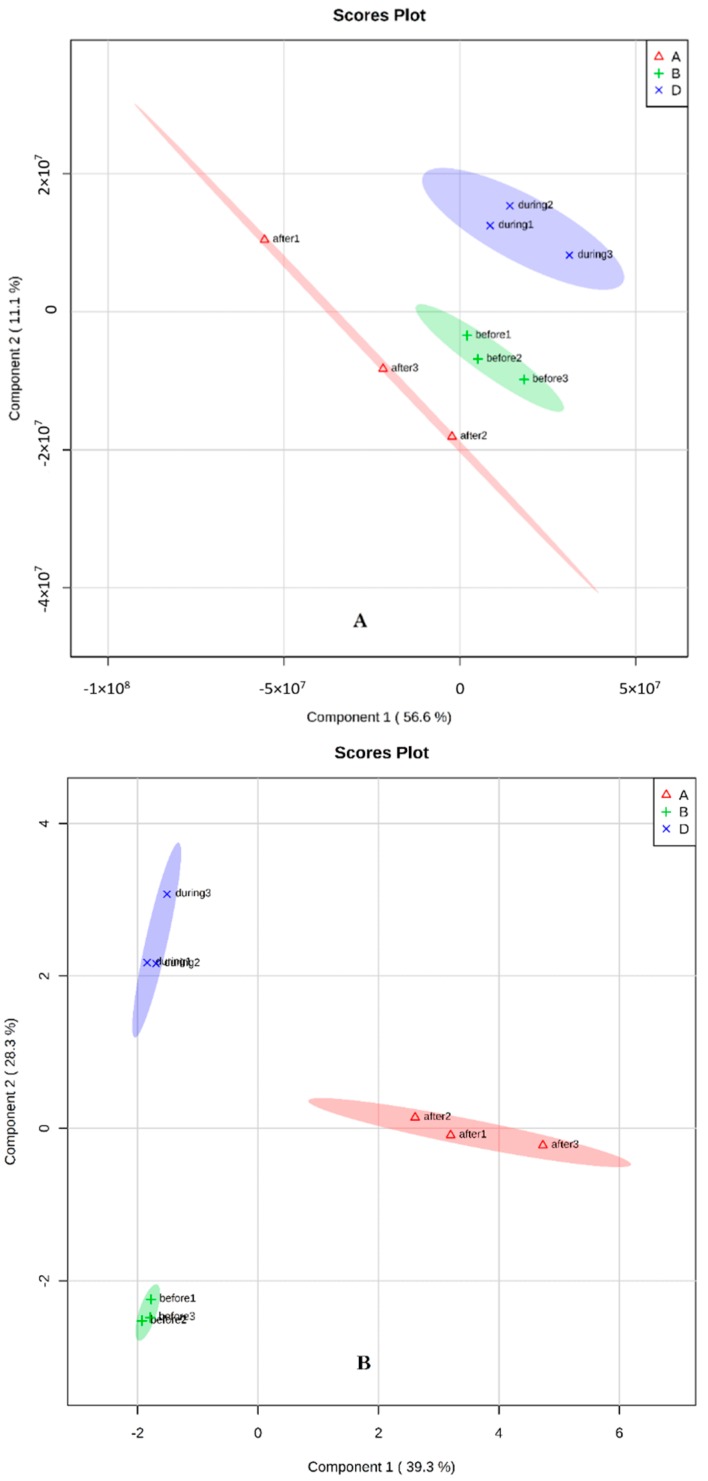
Score plots from Sparse Partial Least Squares-Discriminant Analysis (sPLS-DA) analyzed based on the total peak area obtained from GC-MS data of DI-SPME samples from three different mating stages of medfly: +, before mating; ×, during mating; ∆, after mating using two solvents (**A**) and (**B**). Three symbols in each group mean n = 3 biological replicates.

**Figure 2:**
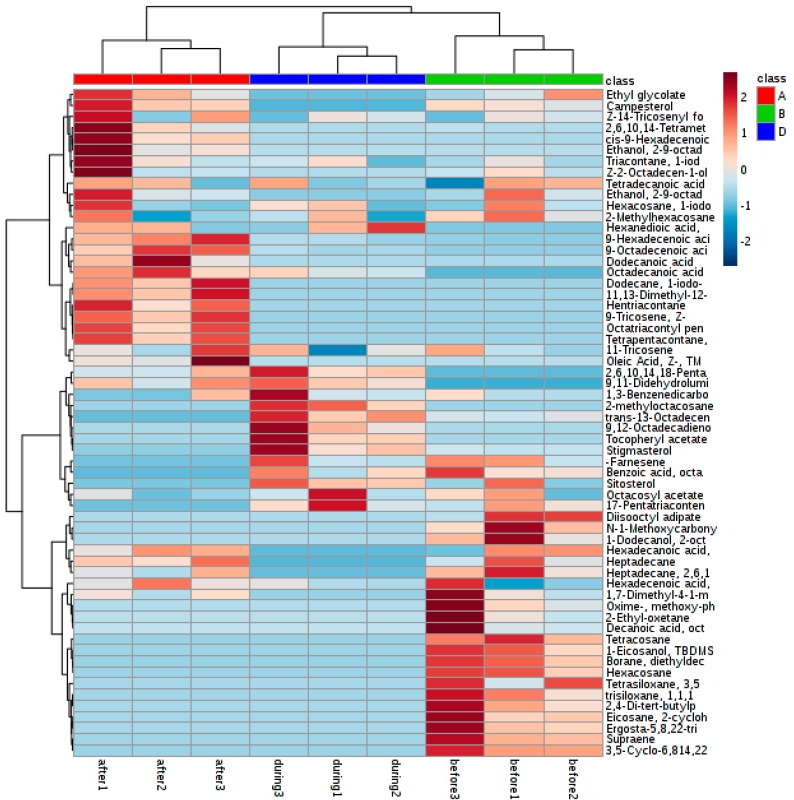
Heat map showing the changes of abundance values normalized to the compounds that are significantly influenced by extraction solvent and the time of insect sampling during mating stage. Three symbols in each group mean n=3 biological replicates.

**Figure 3:**
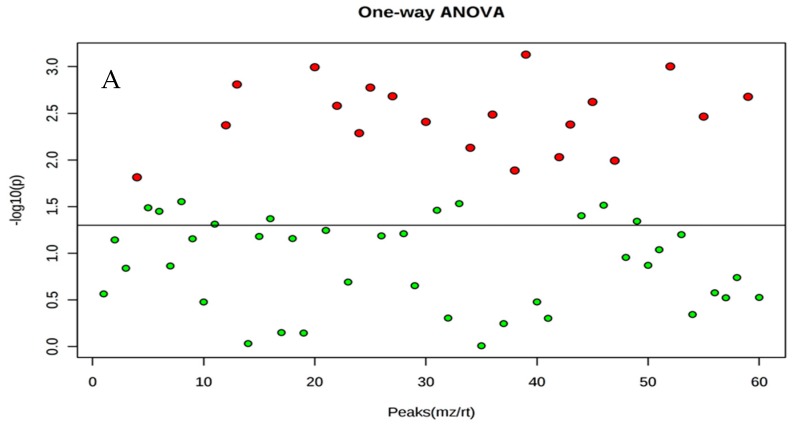

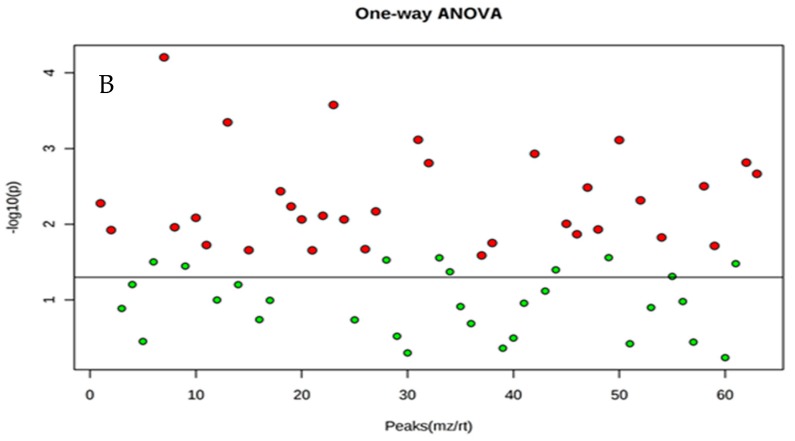
Red points represent significant compounds from the first solvent (**A**) and from the second solvent (**B**). Green points (**A**) and (**B**) are not significant. Each point represent three biological replicates.

The example used is a GC-coupled MS-based metabolomics study in extracted samples where two mating time lines are compared between extract solvents. The two principal components were plotted: the first solvent extraction had 56% and 11.1%, and the second extraction had 39.3% and 28.3% (Figure 1). The heat map showed a clear difference between the samples, particularly during and after mating stage (Figure 2 and Figure 3).

**Figure 4:**
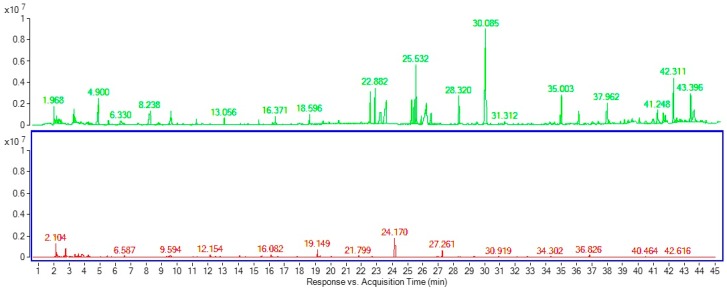
Chromatograms obtained after separation of compounds using DI-SPME and HS-SPME.

Comparing the HS-SPME, compounds including: 2,3-hexanedione, *o*-dimethylbenzene, nonane, 2,3,4-trithiapentane, octanal, acetophenone, 2,6-dimethyl-(*E*,*Z*)-2,4,6-octatriene, 1H-pyrrole-2-carboxylic acid, 2,6,10-trimethyltridecane, dimethyl phthalate; farnesene, (*E*)-γ-bisabolene, 5-phenyl- undecane, carboric acid 2-ethylhexyl octyl ester, 2-ethylhexyl octyl ester; and 5-dodecyldihydro-2(3*H*)-furanone, were detected from medfly adults during mating stage (Table 3, Figure 4). Al-khshemawee et al. [7] reported that the compounds acetoin, 2,3-hexanedione, hexaldehyde, 4-hydroxybutanoic acid, 2,3,4- trithiapentane and octanal were identified from medfly adults using HS-SPME. Jacobson et al. [25] used HS-SPME to identify the pheromones from medfly adults. They found that methyl (*E*)-6-nonenoate and (*E*)-6-nonen-1-ol were the main compounds. Baker et al. [14] studied the volatile compounds emitted by sexually mature male Mediterranean fruit flies. They have been identified the key component involved in the sexual attraction of virgin female flies to males demonstrated to be the novel sex pheromone 3,4-dihydro-2*H*-pyrrole. Cossé et al. [26] reported that the male-produced volatiles eliciting responses from female were ethyl (*E*)-3-octenoate, geranyl acetate, (*E*,*E*)-α-farnesene, linalool, and indole, while Jang et al. [12] found and identified five major component groups that included ethyl hexenoates, hexanoates, methyl octenoates, monoterpenes and ketones. 

Identifications are based on comparisons of both mass spectral data and GC retention indices with those of authentic reference compounds. Several components remain unidentified. Most of the unidentified run components were present at low concentrations, and were therefore thought to be contaminants. Some compounds were presented before mating, but they were missing during the mating stage. Some chemicals, were increased and some decreased within the mating stages (Table 1 and Table 2). McDonald [27] reported that medfly males are stimulated to more frequent episodes of calling activity, when they are able to detect the presence of other medfly males. However, this interaction to visual and acoustic cues rather than to chemical communication. Jacobson et al. [25] and Ohinata et al. [28] studied which components are necessary to trigger an attractive response from female flies. This has been addressed to varying degrees except the present one, which is primarily a qualitative and semi-quantitative examination of the male emission complex. Ongoing laboratory evaluations of the major pheromone components identified indicate that many compounds contribute differentially, but synergistically to the pheromone’s attractiveness for the female medflies. Other intermediate to low-concentration components may also be required to attain full parity with calling males. Flath et al. [29]; Al-khshemawee et al. [30] reported that three different medfly ages (5–6, 11–12, and 20–21 days old), and early-, mid-, and late-morning samples were used to collect volatiles. Thirty-two components were identified. However, propan-2-ol, hexanal, phenol, (*Z*,*E*)-α-farnesene, prop-2-yl-(*E*)-3-octenoate, ethyl (*E*)-2-octenoate, and propyl (*E*)-3-octenoate had been only partially identified in an earlier study. Quantitatively, ethyl acetate, 1-pyrroline, ethyl (*E*)-3-octenoate, geranyl acetate, and α-farnesene were the most abundant emission components from 5–6- and 11–12-day from old flies. The major compound for al fly ages was (2*S*)-2-hexenoic acid. Shelly [31] investigated the influence of α-copaene-containing plants on the mating system of *C. capitata* and the possibility of using attractants in prerelease exposure of males to increase the effectiveness of sterile insect release programs. Mature males were exposed to 20 μL of the attractant over a 6-h period and then were held for 2 d before testing. In field-cage trials, treated males (exposed to attractants) obtained significantly more matings than control males (no exposure) for all three substances. The potential exists for the development of an effective and useful female attractant, especially if essential components and their optimum release rates can be pinpointed and reproduced.

DI-SPME-GC-FID was first reported in an analysis of 13 commonly known benzodiazepines in urine [14]. The same group reported a modification of the method to analyze the hydrolysis of benzodiazepines from benzophenones extraction [32]. DI-SPME has been reported for quantitative analysis of biological samples including plant tissues [33], pesticides [4,34], milk [35], pharmaceuticals [36], wine [37] and water [38]. Myung et al. [39] optimized the DI extraction in blood samples for sorption of 1-octanol. Frérot et al. [40] used an organic solvent to soak or wash SPME in detected pheromones from the female abdominal tip of the Lepidopteran *Sesamia nonagrioides*. The pheromones of *Metamasius hemipterus* (Coleoptera) were sampled using SPME and compared to typical analytical methodologies. The SPME technique was shown to be cheaper, easier, faster and more reproducible [41]. SPME has been used to analyze cuticular hydrocarbons from ants [42]. DI-SPME has been used with pentane or hexane to analyze signaling chemicals and long-chain hydrocarbons from different parts of wasps’ bodies [43]. The SPME technique has also been used to detect long-chain free fatty acids from insect exocrine glands, using a GC-MS [44]. Long chain fatty acids, such as oleic, palmitic, stearic, linoleic, and palmitoleic acids have been found in the exocrine secretions and cuticular extracts of many insects [45]. These compounds are important in intermediates and metabolites of biological pathways, and analytical techniques to study these compounds are of interest [46]. Filho et al. [47] showed that DI-SPME is more sensitive than HS-SPME, and it is thus the method of choice for the analysis of clean aqueous samples. The two extraction modes were evaluated and, despite being less sensitive than HS-SPME in the case of the more volatile compounds, DI-SPME mode successfully extracted 16 pesticides, while HS-SPME was able to extract only 12 compounds. 

## 3. Materials and Methods

### 3.1. Insect Rearing 

A medfly colony was obtained from the Department of Primary Industries and Regional Development (DPIRD), and flies were reared in the Post-harvest Biosecurity and Food Safety Laboratory at Murdoch University (Perth, Western Australia). All the flies were reared under the following conditions: temperature = 23 ± 2 °C, relative humidity = 75 ± 5%, and light: dark cycle = 12:12-h [48]. Adults were placed in screen cages (40 cm cubes), each containing medfly food made from crystaline sugar (Bidvest, Sydney, Australia) and yeast hydrolysate (Australian Biosearch, Sydney, Australia) at a ratio of 4:1, and 50 mL water. Approximately 10–12 days after adult emergence from pupae and mating, eggs were collected each day. These were deposited on a mesh side of the cage and fell into a water tray kept adjacent to the cage.

### 3.2. DI-SPME Conditions

A GC-MS 7890B gas chromatograph equipped with a 5977B MSD mass spectrometer (Agilent Technologies, Santa Clara, CA, USA), with an Agilent HP-5MS column (30 m, 0.25 µm, 0.25 μm film thickness) was used in the experiments. The carrier gas used was helium at 99.999% (BOC, Sydney, Australia). The conditions for the GC-MS were as follows: injector port temperature of 270 °C; initial oven temperature of 60 °C, which increased to 320 °C (at 5 °C/min); MS Quad at 150 °C; MS source at 230 °C; pressure at 10.629 psi. The flow rate was 1.2 mL/min; the splitless was 30 mL/min at 1.0 min. The total run time was 45.40 min. 

Standard *n*-alkane (C_7_-C_30_) reference material containing 1000 μg/mL of each component (decane, docosane, dodecane, eicosane, heneicosane, heptacosane, heptadecane, hexacosane, hexadecane, heptane, nonacosane, nonadecane, nonane, octacosane, octadecane, octane, pentacosane, pentadecane, tetracosane, tetradecane, triacontane, tricosane, tridecane and undecane) in hexane was purchased from Sigma-Aldrich (catalogue number 49451-U; Castle Hill, NSW, Australia), as was *n*-hexane (95%, catalogue number 270504-2L).

### 3.3. DI-SPME Procedure and Sampling Setup

SPME fiber 50/30 μm with Carboxen/DVB/PDMS (Sigma-Aldrich, Bellefonte, PA, USA) coating was inserted into extracted samples. SPME in the samples was conditioned at room temperature (25 ± 5 °C) for 16 h with a sampling depth of 3 cm. The DI-SPME extraction was carried out by immersing the fiber (length: 1.3 cm) into the extracted solution. After extraction for 16 h sealing time, the fiber was withdrawn into the needle, removed from the vial and immediately introduced into the GC injector port for thermal desorption. Samples in triplicate were used for extraction. For sample preparation, adult medflies (0.05 g) were taken before, during and after mating stages. Insects were grinded using tissuelyser at 270 rpm for 2 min. Two extraction solvents, acetonitrile/water (1:1) and methanol/acetonitrile/water (2:2:1) (CAS: 67-56-1, UN1230, Thermo Fisher Scientific, Perth, Australia), were used to extract the samples. Extraction solvent (1 mL) was added to the samples, and centrifuged at 2000 rpm for 5 min. The extraction samples were transferred to a 2 mL analytical vial. SPME was inserted directly into the vial for 16 h at room temperature. Then, the DI-SPME was analyzed using a GC-MS for 15 min desorption time. The samples were analyzed in biological triplicates. 

### 3.4. Statistical Analysis

To observe the impact of observations, principal component analysis (PCA) with the correlation matrix method was used for statistical analysis using the online MetaboAnalyst 3.0 (2017) (Bellevue, Quebec, USA) tool, a comprehensive online tool for metabolomics analysis and interpretation. PCA was used to transfer the original data onto new axes where principal components corresponded to significant information represented by the original data. Three principal components are chosen from the result of PCA and sPLS-DA analysis based on Xia and Wishart [49]. The plots classifier was used to integrate the two components obtained from PCA and produce a segmented image. Since the heatmap centers were chosen randomly in the original means and the obtained results can be different for every run of the algorithm, the overall classification accuracies were averaged over different data. 

## 4. Conclusions

In this study, two DI-SPME extraction solvents for were used at three different stages of the medfly adult life. The first extraction solvent was acetonitrile/water, and the second solvent was methanol/acetonitrile/water. Samples were collected before, during and after mating. This study compared these extraction solvents based on the metabolites extracted. The GC-MS analytical data showed a wide spectrum of compounds and DI-SPME sampling was developed to identify these compounds from medfly extracts. These results indicate that DI-SPME coupled with the GC-MS could be performed successfully on medfly extracts. Using DI-SPME with GC analysis of extracts, high sensitivity and good repeatability were obtained. This work is an example of the application of DI-SPME-GC in the analysis of complex samples and provides a way in which to prepare the samples of SPME coatings. Further development of DI-SPME is promising, and may provide an efficient extraction technique for biological samples.

## Figures and Tables

**Table 1:** Significant compounds peak area (one unit corresponds to a 10^4^ area) detected at three mating stages of medfly by DI-SPME-GC-MS in acetonitrile/water solvent.

Compounds	RI ^a^	RT ^b^	Mating Stages			*p* Value	FDR ^d^
			Before	During	After		
Dodecanoic acid	1572.6	17.342	N.D ^c^	N.D ^c^	104.884	0.003	0.015
1-Iodododecane	1716.2	19.656	N.D ^c^	N.D ^c^	108.690	0.002	0.014
Tetracosane	2078.5	25.429	110.994	N.D ^c^	N.D ^c^	0.001	0.014
*trans*-13-Octadecenoic acid	2122.7	26.132	361.845	980.758	N.D ^c^	0.002	0.014
(*Z*)-Oleic acid	2130.2	26.249	N.D ^c^	N.D ^c^	618.801	6.670	0.014
Octadecanoic acid	2142.1	26.434	N.D ^c^	N.D ^c^	209.611	0.005	0.018
9-(*Z*)-Tricosene	2244.1	28.066	N.D ^c^	N.D ^c^	211.876	0.001	0.014
Hexacosane	2268.5	28.452	96.895	N.D ^c^	N.D ^c^	0.002	0.014
1-Eicosanol, TBDMS derivative	2327.8	30.144	44.947	N.D ^c^	N.D ^c^	0.003	0.015
Supraene	2748.8	36.122	434.511	N.D ^c^	N.D ^c^	0.007	0.024
2-Methyloctacosane	2785.6	36.698	N.D ^c^	44.210	N.D ^c^	0.003	0.015
Diethyldecyloxyborane	2831.5	37.430	66.238	N.D ^c^	N.D ^c^	0.001	0.014
3,5-Cyclo-6,814,22-ergostatriene	2873.7	38.086	64.498	N.D ^c^	N.D ^c^	7.440	0.014
Hentriacontane	2969.3	39.616	N.D ^c^	N.D ^c^	403.452	0.009	0.024
Octatriacontyl pentafluoropropionate	2991.1	39.964	N.D ^c^	N.D ^c^	70.866	0.004	0.015
1,54-Dibromotetrapentacontane	3017.3	40.379	N.D ^c^	N.D ^c^	72.014	0.002	0.014
9-(*Z*)-Hexadecenoic acid hexadecyl ester	3131.3	42.196	55.305	214.519	1583.587	9.960	0.014
11,13-Dimethyl-12-tetradecen-1-acetate	3137.0	42.888	N.D ^c^	N.D ^c^	139.731	0.003	0.015
9-(*E*)-Octadecenoic acid	3251.9	44.119	N.D ^c^	76.668	600.066	0.002	0.014

^a^ RI id retention index; ^b^ RT is retention times; ^c^ N.D is not detected; ^d^ FDR is false discovery rate of data. Each number represent the mean of three biological replicates.

**Table 2:** Significant compounds peak area (one unit corresponds to a 104 area) detected at three mating periods of medfly by DI-SPME-GC-MS in methanol/acetonitrile/water solvent.

Name	RI ^a^	RT ^b^	Mating Stages			*p* Value	FDR ^d^
			Before	During	After		
N-methyleneethanamine	749.4	1.312	162.767	N.D ^c^	N.D ^c^	0.005	0.023
Ethyl glycolate	780.5	1.954	N.D ^c^	N.D ^c^	259.978	0.011	0.031
2,5-Dihydroxybenzaldehyde	1123.1	8.720	100.924	N.D ^c^	N.D^c^	6.250	0.003
Acetic acid 2-propyltetrahydropyran-3-yl ester	1181.3	9.551	N.D ^c^	283.245	N.D ^c^	0.010	0.031
Diclofop-methyl	1266.7	11.602	N.D ^c^	78.171	N.D ^c^	0.008	0.027
1,2-Dihydro-2,2,4-trimethylquinoline	1452.6	15.297	43.9242	119.575	N.D ^c^	0.018	0.041
α-Farnesene	1513.7	16.367	281.554	190.567	N.D ^c^	0.001	0.009
Decanoic acid octyl ester	1650.5	18.601	116.138	N.D ^c^	N.D ^c^	0.021	0.043
Dodecane, 1-iodo-	1716.2	19.656	N.D ^c^	N.D ^c^	108.690	0.003	0.019
Tetradecanoic acid	1765.4	20.432	70.0986	N.D ^c^	88.350	0.005	0.024
2,6,10,15-Tetramethylheptadecane	1892.7	22.466	52.3699	1066.241	176.519	0.008	0.027
Hexadecanoic acid methyl ester	1917.5	22.861	759.908	1283.292	N.D ^c^	0.022	0.043
Hexadecanoic acid pyrrolidide	1937.7	23.182	N.D ^c^	1168.109	N.D ^c^	0.000	0.008
9-Hexadecenoic acid pyrrolidide	1944.1	23.182	382.040	N.D ^c^	757.991	0.001	0.027
1-Piperidin-1-yl-hexadecan-1-one	1958.6	23.518	982.573	N.D ^c^	1095.741	0.008	0.027
9,12-(*Z*,*Z*)-Octadecadienoic acid	2078.2	25.428	356.887	2684.126	N.D ^c^	0.021	0.043
Methyl stearate	2105.0	25.849	226.924	294.663	N.D ^c^	0.006	0.026
Heneicosyl acetate	2181.3	27.073	56.3354	N.D ^c^	N.D ^c^	0.001	0.009
9-(*Z*)-Tricosene	2244.1	28.066	N.D ^c^	N.D ^c^	209.611	0.001	0.012
Trimesitylborane	2672.6	34.89	316.653	N.D ^c^	1398.338	0.025	0.049
1,4-Benzenedicarboxylic acid bis-2-ethylhexyl ester	2679.7	35.001	N.D ^c^	1345.4	N.D ^c^	0.017	0.041
9,11-Didehydrolumisterol acetate	2865.1	37.957	652.982	N.D ^c^	495.747	0.001	0.012
Stigmasta-3,5-diene	2967.1	39.578	N.D ^c^	225.929		0.009	0.029
β-Sitosterol acetate	2968.5	39.601	86.1762	N.D ^c^	403.452	0.013	0.034
Octatriacontyl pentafluoropropionate	2991.1	39.964	N.D ^c^	N.D ^c^	70.866	0.003	0.018
α-Tocopheryl acetate	2995.1	40.029	152.892	N.D ^c^	N.D ^c^	0.011	0.031
3β,22(*E*)-Ergosta-5,8,22-trien-3-ol	3055.5	40.981	N.D ^c^	217.940	N.D ^c^	0.007	0.009
3-Stigmasta-5,22-dien-3-ol acetate	3094.1	41.611	259.867	231.980	N.D ^c^	0.004	0.023
9-Hexadecenoic acid hexadecyl ester	3131.3	42.196	N.D ^c^	N.D ^c^	509.690	0.014	0.036
11,13-Dimethyl-12-tetradecen-1-ol acetate	3137.0	42.888	N.D ^c^	N.D ^c^	176.459	0.003	0.018
Palmitoleic acid	3189.3	43.124	N.D ^c^	N.D ^c^	139.731	0.019	0.041
9-(*E*)-Octadecenoic acid,	3251.9	44.119	N.D ^c^	N.D ^c^	600.066	0.001	0.012
9-Hexadecenoic acid octadecyl ester	3257.9	44.219	303.882	N.D ^c^	1583.587	0.002	0.015

^a^ RI is retention index; ^b^ RT is retention times; ^c^ N.D is not detected; ^d^ FDR is false discovery rate of data. Each number represent the mean of three biological replicates.

**Table 3:** Compounds identified from the adult stage of the medfly (one unit corresponds to a 10^5^ area) determined by GC–MS using HS-SPME.

RT ^a^	Compounds ^b^	RI ^c^	Peak Area
3.61	Acetoin	717	97.830
4.21	Toluene	755	20.493
5.54	Hexaldehyde	769	9.270
7.87	*o*-Dimethylbenzene	862	5.312
8.29	Nonane	900	4.095
9.67	4-Hydroxybutanoic acid	933	8.433
11.29	2,3,4-Trithiapentane	943	1.765
12.19	2,7-dimethyloctane	964	46.140
12.79	Octanal	982	2.035
13.75	4-Methyl-5-hexen-4-olide	996	3.624
14.57	Acetophenone	1049	0.851
15.52	3,3-Dimethylstyrene	1099	2.474
16.06	Cosmene	1134	5.422
16.52	2,6-Dimethyl-(*E*,*Z*)-2,4,6-octatriene	1292	4.970
19.08	2,6-Dimethylundecane	1214	1.554
19.26	1*H*-Pyrrole-2-carboxylic acid	1276	2.032
21.89	Tridecane	1300	2.965
22.66	2,6,10-Trimethyltridecane	1467	1.025
25.89	Dimethyl phthalate	1440	1.275
26.54	Cuparene	1496	1.677
27.01	Farnesene	1499	0.871
28.25	(*E*)-γ-Bisabolene	1523	1.849
30.27	5-Phenylundecane	1626	0.862
32.81	Tetradecanoic acid	1748	1.287
34.27	Carboric acid 2-ethylhexyl octyl ester	1857	0.422
36.82	*n*-Hexadecanoic acid	1968	2.129
38.56	5-Dodecyldihydro-2(3*H*)-furanone	2120	0.382
40.07	Octadecanoic acid	2187	1.743

^a^ RT: retention time (min); ^b^ Compounds, name of compounds detected by GC-MS; ^c^ RI: retention indices. Each number of peak area represent three biological replicates.
